# Poly[di­ammonium [di­aqua­(μ_7_-benzene-1,2,3,4,5,6-hexa­carboxyl­ato)tetra­oxido­diuranium(VI)]]

**DOI:** 10.1107/S1600536814006047

**Published:** 2014-03-22

**Authors:** Paula M. Cantos, Christopher L. Cahill

**Affiliations:** a Department of Chemistry, The George Washington University, 725 21st St NW, Washington, DC 20052, USA

## Abstract

Uranyl-carboxyl­ate hybrid materials dominate the catalog of uranyl compounds owing in part to the affinity between COO^−^ functional groups and UO_2_^2+^. Polycarboxyl­ate organic ligands may present a degree of steric hindrance and could thus influence the resulting uranyl topology. Single crystals of the title compound, {(NH_4_)_2_[(UO_2_)_2_(C_12_O_12_)(H_2_O)_2_]}_*n*_, were synthesized hydro­thermally as a result of reacting uranyl nitrate with benzene-1,2,3,4,5,6-hexa­carb­oxy­lic acid (mellitic acid). The structure is comprised of a single unique monomeric uranyl cation adopting a penta­gonal bipyramidal geometry. The uranyl coordination sphere is composed of four O atoms originating from one half of a fully deprotonated mellitic acid ligand and a single water mol­ecule. The observed axial U—O bonds display an average distance of 1.765 (8) Å, whereas equatorial O atoms are found at an average distance of 2.40 (5) Å. All uranium–oxygen bond lengths are in good agreement with literature values. Furthermore, the coordin­ation between the uranyl penta­gonal bipyramids and the mellitic acid anion constructs a three-dimensional anionic framework which is charge-balanced with ammonium cations. Additional stabilization of the structure is provided by O—H⋯O and N—H⋯O hydrogen bonding inter­actions between the components.

## Related literature   

The background literature for uranyl aromatic, carboxyl­ate coordination polymers is extensive: Go *et al.* (2007 ▶); Andrews & Cahill (2012 ▶); Frisch & Cahill (2006 ▶); Rowland & Cahill (2010 ▶); Couston *et al.* (1995 ▶); Severance *et al.* (2011 ▶); Mihalcea *et al.* (2012 ▶); Thuery (2009 ▶); Leciejewicz *et al.* (1995 ▶). For related uranyl mellitic complexes, see: Volkringer *et al.* (2012 ▶). For *f*-block homo- and heterometallic mellitic acid compounds, see: Li *et al.* (2006 ▶); Tang *et al.* (2008 ▶); Taylor *et al.* (2008 ▶); Chui *et al.* (2001 ▶); Han *et al.* (2012 ▶); Mihalcea *et al.* (2012 ▶); Volkringer *et al.* (2012 ▶). For typical U=O bond lengths, see: Burns (2005 ▶). 
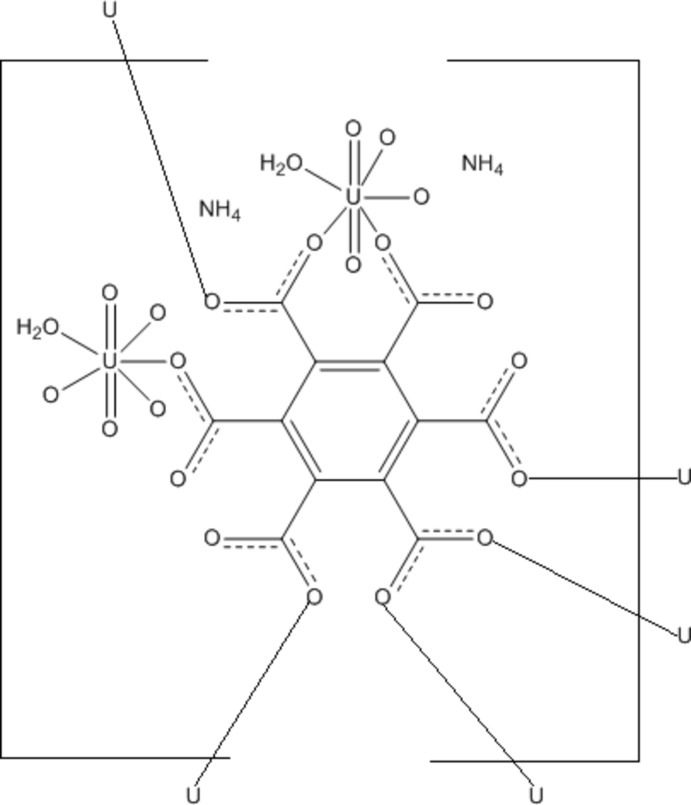


## Experimental   

### 

#### Crystal data  

(NH_4_)_2_[(UO_2_)_2_(C_12_O_12_)(H_2_O)_2_]
*M* *_r_* = 948.29
Monoclinic, 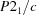

*a* = 8.0083 (4) Å
*b* = 10.2948 (6) Å
*c* = 11.7481 (6) Å
β = 99.733 (1)°
*V* = 954.62 (9) Å^3^
*Z* = 2
Mo *K*α radiation
μ = 17.05 mm^−1^
*T* = 100 K
0.4 × 0.3 × 0.2 mm

#### Data collection  

Bruker APEXII CCD diffractometer
Absorption correction: multi-scan (*SADABS*; Sheldrick, 1999 ▶) *T*_min_ = 0.467, *T*_max_ = 0.746
18160 measured reflections
2912 independent reflections
2398 reflections with *I* > 2σ(*I*)
*R*_int_ = 0.036

#### Refinement  

*R*[*F* ^2^ > 2σ(*F* ^2^)] = 0.017
*wR*(*F* ^2^) = 0.035
*S* = 1.04
2698 reflections
178 parameters
All H-atom parameters refined
Δρ_max_ = 1.02 e Å^−3^
Δρ_min_ = −0.98 e Å^−3^

### 

Data collection: *APEX2* (Bruker, 2008 ▶); cell refinement: *SAINT* (Bruker, 2008 ▶); data reduction: *SAINT*; program(s) used to solve structure: *SHELXS97* (Sheldrick, 2008 ▶); program(s) used to refine structure: *SHELXL97* (Sheldrick, 2008 ▶); molecular graphics: *CrystalMaker* (CrystalMaker, 2009 ▶) and *ORTEP-3* (Burnett & Johnson 1996 ▶); software used to prepare material for publication: *WinGX* (Farrugia, 2012 ▶).

## Supplementary Material

Crystal structure: contains datablock(s) I, publication_text. DOI: 10.1107/S1600536814006047/gg2133sup1.cif

Structure factors: contains datablock(s) I. DOI: 10.1107/S1600536814006047/gg2133Isup2.hkl

CCDC reference: 992448

Additional supporting information:  crystallographic information; 3D view; checkCIF report

## Figures and Tables

**Table 1:** Hydrogen-bond geometry (Å, °)

*D*—H⋯*A*	*D*—H	H⋯*A*	*D*⋯*A*	*D*—H⋯*A*
O9—H6⋯O4^i^	0.74 (5)	2.02 (5)	2.700 (3)	152 (5)
O9—H5⋯O6^ii^	0.91 (6)	1.88 (5)	2.744 (3)	158 (4)
O9—H5⋯O7	0.91 (6)	2.38 (5)	2.884 (3)	115 (4)
N1—H3⋯O2^iii^	0.81 (5)	2.12 (5)	2.908 (4)	165 (5)
N1—H1⋯O5^iv^	0.85 (4)	2.36 (4)	2.990 (4)	131 (3)
N1—H1⋯O6^v^	0.85 (4)	2.29 (4)	2.905 (4)	130 (3)
N1—H4⋯O7	0.94 (5)	1.94 (5)	2.851 (4)	162 (5)

Symmetry codes: (i) 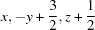; (ii) 
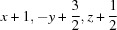
; (iii) 
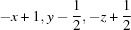
; (iv) 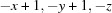; (v) 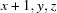.

**Fig. 1.**
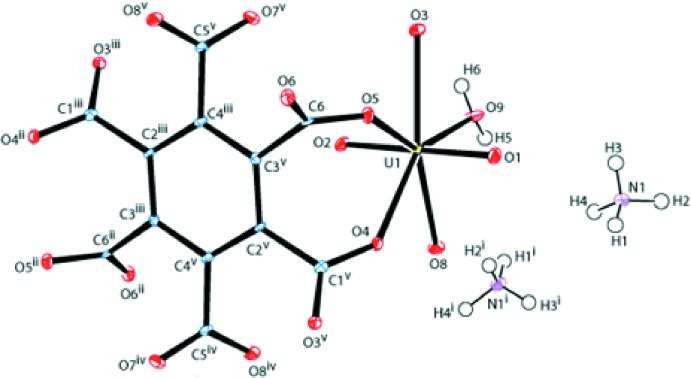
The ORTEP representation of an asymmetric unit of (NH4)2[(UO2)2(C12O12)(H2O)2]. The ellipsoids are shown at the 50% level and the hydrogen atoms have been removed for clarity. [Symmetry codes: (i) x,-y + 3/2,z - 1/2; (ii) x,-y + 3/2,z + 1/2; (iii) -x,-y + 1,-z + 1; (iv) -x + 1,y + 1/2,-z + 1/2; and (v) -x + 1,y - 1/2,-z + 1/2].

**Fig. 2.**
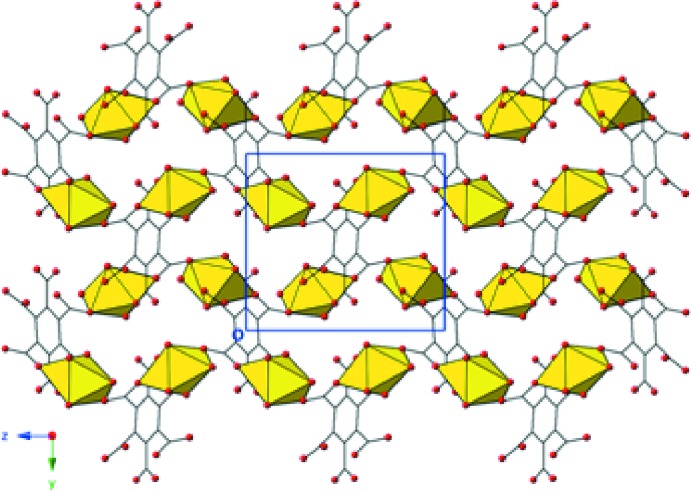
Polyhedral representation of the channels down the [100] in 1. Yellow polyhedra represent uranium atoms; and red spheres, oxygen atoms. Hydrogen atoms and charge balancing NH4 molecules have been removed for clarity.

## supplementary crystallographic information

### 1. Comment

The portfolio of uranyl hybrid materials displays diverse architectures, which may be attributed to the modification of synthetic parameters, especially varying the organic ligand. The literature provides prior studies focused on the reaction of the uranyl cation with a series of related organic ligands, typically decorated with carboxylate functional groups, in order to observe an influence on overall topology. The position of carboxylic acids on an aromatic ligand, such as those on mellitic acid, allows one to investigate the steric influence of multiple functional groups on the local and the global architecture of a UO_2_^2+^ hybrid material. Indeed, other f-block homo- and heterometallic mellitic acid compounds have been reported in the literature. Li *et al.*, (2006); Tang *et al.*, (2008); Taylor *et al.*, (2008); Chui *et al.*, (2001); Han *et al.*, (2012); Mihalcea *et al.*, (2012); Volkringer *et al.*, (2012).

The title coordination polymer was synthesized hydrothermally and contains a pentagonal monomer (U1) of the uranyl mellitic hybrid material contains typical U=O axial bond distances to O1 and O2 (1.760 (2) and 1.771 (2) Å, respectively) Burns (2005). Three mellitic acid molecules bind to U1: O3 and O8 bond in a monodentate fashion, while O4 and O5 participate in a pseudo-bidentate mode. An oxygen atom (O9) from a water molecule completes the local coordination sphere of U1 yielding a distance of 2.452 (2) Å. The rotation of carboxylate functional groups are a direct consequence of sterics within (NH_4_)_2_[(UO_2_)_2_(C_12_O_12_)(H_2_O)_2_]. The proximity of adjacent carboxylate groups O5—C6—O6 and O4—C1—O3 located on the benzene ring prompts a rotation of these functional groups (yielding torsion angles of 50.62° and 67.27°, respectively), thus connecting both to U1 while O4—C1—O3 further bonds to U1^i^. Similarly, O8^ii^—C5^ii^—O7^ii^ bonds to U1^ii^ and extends the formation of channels along the [100] direction as seen in Figure 2.

### 2. Experimental

Caution! Whereas the uranium oxynitrate hexahydrate, (UO_2_)(NO_3_)_2_·H_2_O, used in this investigation contains depleted uranium, standard precautions for handling radioactive substances should be followed. Uranium nitrate was recrystallized from a mixture of uranyl nitrate and uranyl oxide dissolved in concentrated nitric acid. Powder X-ray diffraction confirmed the formation of uranium oxinitrate hexahydrate. (PDF-#27–0936). Mellitic acid was commercially available and used without any further purification. Uranium oxynitrate hexahydrate (0.146 g, 0.29 mmol), mellitic acid (0.050 g, 0.14 mmol), and deionized water (1.5 ml, 83.2 mmol) were placed into a 23 ml Teflon-lined Parr bomb. Concentrated ammonium hydroxide was used to adjust the pH (pHi = 4.38). The vessel was sealed and heated statically at 150°C for three days. Yellow prismatic crystals were obtained. Phase purity was confirmed *via* powder X-ray diffraction data patterns. Calculated and observed elemental analysis results (Galbraith Laboratories, Knoxville, Tennessee, USA) of 1 agreed, confirming the contents of the material [observed (calculated): C 1.47% (1.51%), H < 0.5% (0.10%), N < 0.5% (0.25%)].

### 3. Refinement

The hydrogen atoms on ammonium and water molecules were located in a difference Fourier map. No hydrogen atoms were located within bonding distance of oxygen atoms O6 and O7 and there was no attempt to calculate positions of riding H atoms on these two oxygen sites. Residual electron density surroudning U1 is noted and the deepest hole is 1.02 Å from U1. This may be considered an artifact of the heavy atom site.

### Crystal data

**Table:**

(NH_4_)_2_[(UO_2_)_2_(C_12_O_12_)(H_2_O)_2_]	*F*(000) = 852
*M**_r_* = 948.29	*D*_x_ = 3.299 Mg m^−^^3^
Monoclinic, *P*2_1_/*c*	Mo *K*α radiation, λ = 0.71073 Å
Hall symbol: -P 2ybc	Cell parameters from 18686 reflections
*a* = 8.0083 (4) Å	θ = 7.0–60.6°
*b* = 10.2948 (6) Å	µ = 17.05 mm^−^^1^
*c* = 11.7481 (6) Å	*T* = 100 K
β = 99.733 (1)°	Rods, yellow
*V* = 954.62 (9) Å^3^	0.4 × 0.3 × 0.2 mm
*Z* = 2	

### Data collection

**Table:**

Bruker APEXII CCD diffractometer	2912 independent reflections
Radiation source: fine-focus sealed tube	2398 reflections with *I* > 2σ(*I*)
Graphite monochromator	*R*_int_ = 0.036
ω scans	θ_max_ = 30.5°, θ_min_ = 2.7°
Absorption correction: multi-scan (*SADABS*; Sheldrick, 1999)	*h* = −11→8
*T*_min_ = 0.467, *T*_max_ = 0.746	*k* = −14→14
18160 measured reflections	*l* = −16→16

### Refinement

**Table:**

Refinement on *F*^2^	Primary atom site location: structure-invariant direct methods
Least-squares matrix: full	Secondary atom site location: difference Fourier map
*R*[*F*^2^ > 2σ(*F*^2^)] = 0.017	Hydrogen site location: inferred from neighbouring sites
*wR*(*F*^2^) = 0.035	All H-atom parameters refined
*S* = 1.04	*w* = 1/[σ^2^(*F*_o_^2^) + (0.0125*P*)^2^ + 1.2966*P*] where *P* = (*F*_o_^2^ + 2*F*_c_^2^)/3
2698 reflections	(Δ/σ)_max_ = 0.001
178 parameters	Δρ_max_ = 1.02 e Å^−^^3^
0 restraints	Δρ_min_ = −0.98 e Å^−^^3^

### Special details

**Table:**

Geometry. All s.u.'s (except the s.u. in the dihedral angle between two l.s. planes) are estimated using the full covariance matrix. The cell s.u.'s are taken into account individually in the estimation of s.u.'s in distances, angles and torsion angles; correlations between s.u.'s in cell parameters are only used when they are defined by crystal symmetry. An approximate (isotropic) treatment of cell s.u.'s is used for estimating s.u.'s involving l.s. planes.
Refinement. Refinement of *F*^2^ against ALL reflections. The weighted *R*-factor *wR* and goodness of fit *S* are based on *F*^2^, conventional *R*-factors *R* are based on *F*, with *F* set to zero for negative *F*^2^. The threshold expression of *F*^2^ > 2σ(*F*^2^) is used only for calculating *R*-factors(gt) *etc*. and is not relevant to the choice of reflections for refinement. *R*-factors based on *F*^2^ are statistically about twice as large as those based on *F*, and *R*- factors based on ALL data will be even larger.

### Fractional atomic coordinates and isotropic or equivalent isotropic displacement parameters (Å^2^)

**Table:**

	*x*	*y*	*z*	*U*_iso_*/*U*_eq_	
U1	0.412520 (13)	0.721169 (10)	0.152007 (9)	0.00526 (4)	
O1	0.5032 (3)	0.5786 (2)	0.10655 (18)	0.0103 (4)	
O2	0.3232 (3)	0.8648 (2)	0.19911 (18)	0.0091 (4)	
O3	0.2643 (3)	0.6031 (2)	0.28641 (18)	0.0082 (4)	
O5	0.1465 (3)	0.6550 (2)	0.05335 (18)	0.0083 (4)	
O4	0.3585 (3)	0.7973 (2)	−0.04722 (18)	0.0096 (4)	
O8	0.6415 (3)	0.8435 (2)	0.10897 (18)	0.0112 (4)	
O9	0.6149 (3)	0.7076 (2)	0.3302 (2)	0.0125 (5)	
O7	0.8885 (3)	0.7764 (2)	0.21059 (19)	0.0112 (4)	
O6	−0.1157 (3)	0.6646 (2)	−0.04255 (18)	0.0093 (4)	
C1	0.2569 (4)	0.6268 (3)	0.3903 (2)	0.0065 (6)	
C2	0.1216 (4)	0.5613 (3)	0.4453 (2)	0.0050 (5)	
C3	0.0179 (4)	0.6342 (3)	0.5063 (2)	0.0054 (5)	
C5	0.8015 (4)	0.8427 (3)	0.1337 (2)	0.0069 (5)	
C4	0.1018 (4)	0.4272 (3)	0.4383 (2)	0.0064 (6)	
C6	0.0162 (4)	0.7181 (3)	0.0055 (2)	0.0065 (5)	
N1	0.8845 (4)	0.5051 (3)	0.1615 (3)	0.0107 (5)	
H1	0.852 (5)	0.507 (4)	0.089 (4)	0.022 (11)*	
H2	0.980 (6)	0.463 (4)	0.185 (4)	0.031 (13)*	
H3	0.813 (6)	0.468 (4)	0.190 (4)	0.030 (13)*	
H4	0.905 (6)	0.590 (5)	0.190 (4)	0.035 (13)*	
H6	0.571 (6)	0.706 (4)	0.381 (4)	0.031 (14)*	
H5	0.716 (7)	0.748 (5)	0.355 (4)	0.041 (14)*	

### Atomic displacement parameters (Å^2^)

**Table:**

	*U*^11^	*U*^22^	*U*^33^	*U*^12^	*U*^13^	*U*^23^
U1	0.00418 (6)	0.00674 (6)	0.00524 (5)	−0.00009 (4)	0.00189 (4)	0.00013 (4)
O1	0.0106 (11)	0.0116 (11)	0.0092 (10)	0.0035 (9)	0.0032 (8)	0.0001 (8)
O2	0.0094 (11)	0.0093 (11)	0.0096 (10)	−0.0007 (8)	0.0045 (8)	−0.0028 (8)
O3	0.0085 (11)	0.0096 (10)	0.0069 (10)	−0.0019 (8)	0.0023 (8)	−0.0015 (8)
O5	0.0059 (10)	0.0079 (10)	0.0106 (10)	0.0008 (8)	−0.0001 (8)	0.0004 (8)
O4	0.0090 (11)	0.0132 (11)	0.0073 (10)	0.0058 (8)	0.0034 (8)	0.0035 (8)
O8	0.0062 (11)	0.0136 (11)	0.0141 (11)	−0.0020 (9)	0.0027 (8)	0.0039 (9)
O9	0.0083 (12)	0.0229 (14)	0.0068 (11)	−0.0051 (10)	0.0026 (9)	0.0000 (9)
O7	0.0112 (11)	0.0108 (11)	0.0118 (11)	0.0005 (9)	0.0029 (9)	0.0057 (9)
O6	0.0091 (11)	0.0083 (10)	0.0103 (10)	0.0009 (9)	0.0011 (8)	0.0003 (9)
C1	0.0055 (14)	0.0061 (14)	0.0081 (13)	0.0026 (11)	0.0014 (11)	0.0018 (10)
C2	0.0024 (13)	0.0100 (14)	0.0027 (12)	−0.0001 (11)	0.0005 (10)	−0.0005 (10)
C3	0.0047 (13)	0.0054 (13)	0.0055 (13)	0.0001 (10)	−0.0005 (10)	−0.0001 (10)
C5	0.0099 (14)	0.0047 (13)	0.0074 (13)	−0.0004 (11)	0.0056 (11)	−0.0023 (11)
C4	0.0040 (14)	0.0094 (14)	0.0061 (13)	0.0015 (11)	0.0016 (10)	−0.0001 (11)
C6	0.0062 (14)	0.0082 (14)	0.0065 (13)	−0.0007 (11)	0.0048 (10)	0.0005 (11)
N1	0.0113 (15)	0.0110 (14)	0.0108 (14)	0.0006 (11)	0.0045 (11)	0.0019 (11)

### Geometric parameters (Å, º)

**Table:**

U1—O1	1.760 (2)	O6—C6	1.240 (4)
U1—O2	1.771 (2)	C1—O4^ii^	1.269 (4)
U1—O5	2.348 (2)	C1—C2	1.510 (4)
U1—O8	2.349 (2)	C2—C4	1.391 (4)
U1—O9	2.425 (2)	C2—C3	1.402 (4)
U1—O4	2.437 (2)	C3—C4^iii^	1.397 (4)
U1—O3	2.452 (2)	C3—C6^ii^	1.520 (4)
O3—C1	1.256 (4)	C5—C4^iv^	1.514 (4)
O5—C6	1.276 (4)	C4—C3^iii^	1.397 (4)
O4—C1^i^	1.269 (3)	C4—C5^v^	1.514 (4)
O8—C5	1.264 (4)	C6—C3^i^	1.520 (4)
O7—C5	1.247 (4)		
			
O1—U1—O2	179.34 (10)	C6—O5—U1	132.53 (19)
O1—U1—O5	89.70 (9)	C1^i^—O4—U1	138.25 (19)
O2—U1—O5	90.90 (9)	C5—O8—U1	138.1 (2)
O1—U1—O8	90.27 (9)	O3—C1—O4^ii^	123.4 (3)
O2—U1—O8	89.48 (9)	O3—C1—C2	119.0 (3)
O5—U1—O8	136.52 (7)	O4^ii^—C1—C2	117.6 (2)
O1—U1—O9	87.94 (9)	C4—C2—C3	119.3 (3)
O2—U1—O9	91.41 (9)	C4—C2—C1	120.1 (3)
O5—U1—O9	145.87 (8)	C3—C2—C1	120.6 (3)
O8—U1—O9	77.55 (8)	C4^iii^—C3—C2	120.5 (3)
O1—U1—O4	89.55 (8)	C4^iii^—C3—C6^ii^	116.7 (2)
O2—U1—O4	90.93 (8)	C2—C3—C6^ii^	122.5 (3)
O5—U1—O4	67.67 (7)	O7—C5—O8	126.2 (3)
O8—U1—O4	68.85 (7)	O7—C5—C4^iv^	116.3 (3)
O9—U1—O4	146.29 (8)	O8—C5—C4^iv^	117.5 (3)
O1—U1—O3	92.95 (9)	C2—C4—C3^iii^	120.1 (3)
O2—U1—O3	86.99 (8)	C2—C4—C5^v^	122.6 (3)
O5—U1—O3	71.12 (7)	C3^iii^—C4—C5^v^	117.2 (3)
O8—U1—O3	152.22 (7)	O6—C6—O5	123.0 (3)
O9—U1—O3	75.01 (8)	O6—C6—C3^i^	117.0 (3)
O4—U1—O3	138.70 (7)	O5—C6—C3^i^	120.0 (3)
C1—O3—U1	129.60 (19)		

Symmetry codes: (i) *x*, −*y*+3/2, *z*−1/2; (ii) *x*, −*y*+3/2, *z*+1/2; (iii) −*x*, −*y*+1, −*z*+1; (iv) −*x*+1, *y*+1/2, −*z*+1/2; (v) −*x*+1, *y*−1/2, −*z*+1/2.

### Hydrogen-bond geometry (Å, º)

**Table:**

*D*—H···*A*	*D*—H	H···*A*	*D*···*A*	*D*—H···*A*
O9—H6···O4^ii^	0.74 (5)	2.02 (5)	2.700 (3)	152 (5)
O9—H5···O6^vi^	0.91 (6)	1.88 (5)	2.744 (3)	158 (4)
O9—H5···O7	0.91 (6)	2.38 (5)	2.884 (3)	115 (4)
N1—H3···O2^v^	0.81 (5)	2.12 (5)	2.908 (4)	165 (5)
N1—H1···O5^vii^	0.85 (4)	2.36 (4)	2.990 (4)	131 (3)
N1—H1···O6^viii^	0.85 (4)	2.29 (4)	2.905 (4)	130 (3)
N1—H4···O7	0.94 (5)	1.94 (5)	2.851 (4)	162 (5)

Symmetry codes: (ii) *x*, −*y*+3/2, *z*+1/2; (v) −*x*+1, *y*−1/2, −*z*+1/2; (vi) *x*+1, −*y*+3/2, *z*+1/2; (vii) −*x*+1, −*y*+1, −*z*; (viii) *x*+1, *y*, *z*.
